# Multivalent next generation influenza virus vaccines protect against seasonal and pre-pandemic viruses

**DOI:** 10.1038/s41598-023-51024-0

**Published:** 2024-01-16

**Authors:** Naoko Uno, Ted M. Ross

**Affiliations:** 1 Center for Vaccines and Immunology, University of Georgia, Athens, GA USA; 2 Department of Infectious Diseases, University of Georgia, Athens, GA USA; 3 Florida Research and Innovation Center, Cleveland Clinic Florida, 9801 SW Discovery Way, Port Saint Lucie, FL 34986 USA; 4 Department of Infection Biology, Lerner Research Institute, Cleveland Clinic, Cleveland, OH USA

**Keywords:** Immunology, Infectious diseases, Vaccines

## Abstract

Each year, new influenza virus vaccine formulations are generated to keep up with continuously circulating and mutating viral variants. A next-generation influenza virus vaccine would provide long-lasting, broadly-reactive immune protection against current and future influenza virus strains for both seasonal and pre-pandemic viruses. Next generation immunogens were designed using computationally optimized broadly reactive antigen (COBRA) methodology to protect against a broad range of strains over numerous seasons. Novel HA and NA amino acid sequences were derived from multilayered consensus sequence alignment for multiple subtypes of influenza. This multivalent formulation was hypothesized to elicit broadly protective immune responses against both seasonal and pre-pandemic influenza viruses. Mice were vaccinated with multivalent mixtures of HA and NA (H1, H2, H3, H5, H7, N1, N2) proteins. Multivalent COBRA vaccinations elicited antibodies that recognized a broad panel of strains and vaccinated mice were protected against viruses representing multiple subtypes. This is a promising candidate for a universal influenza vaccine that elicits protective immune responses against seasonal and pre-pandemic strains over multiple seasons.

## Introduction

Influenza A viruses (IAV) cause severe respiratory disease in people every year resulting in 290,000 to 650,000 deaths worldwide^1^. IAV can be further distinguished into subtypes identified by the hemagglutinin (HA) and neuraminidase (NA) surface glycoproteins^2^. Currently there are 18 identified HAs (H1–H18), divided into two groups^3^ and 11 identified NAs (N1–N11)^4^ with H1N1 and H3N2 strains are currently circulating in humans. However, pre-pandemic zoonotic strains, H2, H5, and H7, all have pandemic potential to transmit from avian or swine reservoirs and then between humans^5^.

Influenza viruses have circulated in humans for centuries, though it was not isolated until the beginning of the twentieth century^6^. Since 1918, there have been four pandemics caused by IAV: 1918 Spanish flu (H1N1), 1957 Asian flu (H2N2), 1968 Hong Kong flu (H3N2), and 2009 Swine flu (H1N1)^7^. The 2009 pandemic virus (clade 6B.1) has since replaced the prior seasonal H1N1 strains and is the current lineage circulating in humans^8,9^. Currently, H3N2 infections are composed of a wider range of strains from two antigenically distinct clades (clades 3C.2a and 3C.3a)^8,10^. H2Nx, H5Nx, and H7Nx pre-pandemic strains have high potential to transmit to humans, causing outbreaks that result in severe disease and death^11^.

The efficacy of influenza virus vaccines vary year to year, from around 10–60%^12^. An ideal universal Influenza vaccine would provide durable protection against circulating strains, seasonal and pre-pandemic^13^. Computationally optimized broadly reactive antigen (COBRA) methodology has been utilized to design HA and NA antigens that protect against a broad breadth of strains for each subtype^14–19^. The correlate of protection for influenza has historically been hemagglutinin inhibition (HAI) titers of antibodies targeting the HA globular head^20^. Neuraminidase inhibition (NAI) titers have also been examined as an independent role in protection against infection^21^. The COBRA methodology aligns multiple layers of consensus sequences to generate a single immunogen which could protect against a large panel of strains for each subtype^22^. Our previous studies showed monovalent COBRA HA or NA vaccinations elicits wider breadth of immune response compared to wild type counterparts.

In this study, mice were vaccinated intramuscularly with multiple COBRA HA and NA immunogens in quadrivalent (H1, H3, N1, N2) or heptavalent (H1, H2, H3, H5, H7, N1, N2) regimens formulated with AddaVax™, an oil-in-water emulsion adjuvant. These vaccines elicited protective immune responses against a wide breadth of seasonal IAV strains. Heptavalent formulations elicited protective immune responses against a wide breadth of pre-pandemic strains as well. Multivalent COBRA HA and NA proteins are promising candidates for a universal, broadly-reactive influenza vaccine.

## Results

### COBRA rHA and rNA elicit antibodies with broadly-reactive HAI activity

The COBRA methodology has been used to design rHA and rNA proteins representing individual subtypes of influenza virus^14–19^. In this study, multivalent vaccine formulations were generated to cover current circulating IAV subtypes (H1N1, H3N2), as well as future pre-pandemic subtypes (H2, H5, and H7) (Table 1). Mice were vaccinated with recombinant proteins in two different formulations: Quadrivalent formulation consisting of COBRA rHA and rNA representing seasonal subtypes (H1, H3, HAs and N1, N2 NAs) and heptavalent formulation consisted of COBRA rHA and rNA representing both seasonal and pre-pandemic subtypes (H1, H2, H3, H5, H7 HAs and N1, N2 NAs).

**Table 1:** COBRA vaccine components.

COBRA	Protein	Description	References
Seasonal			
Y2	H1 HA	Rolling COBRA based on 2016–2019 human strains	^15^
NG2	H3 HA	Rolling COBRA based on 2016–2018 human strains	^18^
N1I	N1 NA	1990–2015 Avian, swine, and human strains	^19^
N2A	N2 NA	Human 1957–2019 and swine 1977–2019 strains	*Submitted*
Pre-pandemic			
Z1	H2 HA	Avian 1960–2020, human 1957–2020, and swine 1970–2020 strains	^17^
IAN8	H5 HA	Rolling COBRA based on 2011–2016 avian and human strains	^16^
Q6	H7 HA	2000–2018 Avian and human strains	^80^

Computationally optimized broadly reactive antigen (COBRA) methodology was used to design HA and NA immunogens, as previously described. The quadrivalent vaccine formulation consist of the seasonal COBRA components and the heptavalent vaccine formulation consists of both the seasonal and pre-pandemic components.

**Figure 1:**
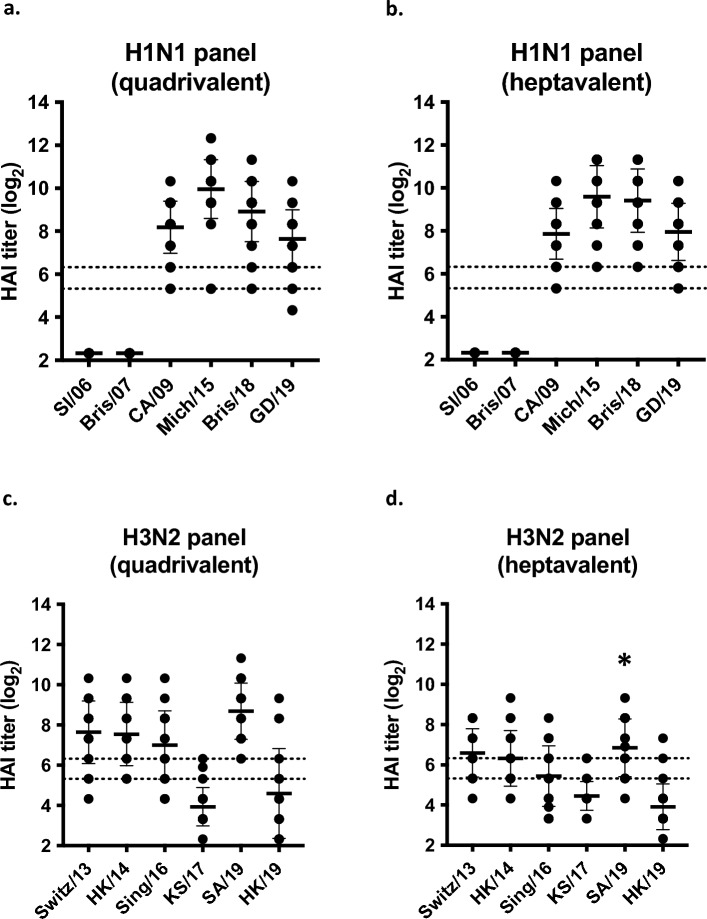
Serum HAI antibody titers for seasonal influenza viruses after vaccination in mice. Immunologically naïve DBA/2J mice were vaccinated intramuscularly (i.m.) three times at four-week intervals with quadrivalent (left) or heptavalent (right) formulations of COBRA recombinant HA and NA proteins, with AddaVax™ as adjuvant. Sera collected two weeks after final vaccination were used for HAI assay against a panel of (**a**, **b**) H1N1 and (**c**, **d**) H3N2 viruses. The virus strains are listed along the x-axis. The y-axis indicates the log_2_ HAI titers for each vaccinated group and presents them as absolute mean values ± SEM. The dotted lines indicate HAI titers ranging from 1:40 (lower line) and 1:80 (upper line). Statistical differences between quadrivalent and heptavalent vaccine groups were analyzed using multiple unpaired *t*-tests by GraphPad Prism 9 software (GraphPad, San Diego, CA, USA). A *p* value of less than 0.05 was defined as statistically significant (*, *p* < 0.05; **, *p* < 0.01; ***, *p* < 0.001; ****, *p* < 0.0001).

Mice vaccinated with either COBRA formulation had statistically similar HAI titers against a panel of H1N1 strains (Fig. 1a,b). Sera collected from COBRA vaccinated mice had robust HAI activity against currently circulating CA/09-like strains (clade 6B.1), but little HAI activity against older historic H1N1 strains (SI/06 and Bris/07). Mice vaccinated with quadrivalent or heptavalent formulations also had antibodies with HAI activity (≥ 1:40 or higher) against four out of six H3N2 strains in the panel (clades 3C.2a and 3C.3a). KS/17 (clade 3c.3a) and HK/19 (subgroup 3c2.a1b/137F) had average antibody titers less than 1:40 (Fig. 1c,d). Quadrivalent vaccine group had statistically higher HAI antibody titers for SA/19 (subgroup 3c2.a1b/131K) than heptavalent, both groups had HAI activity > 1:80.

**Figure 2:**
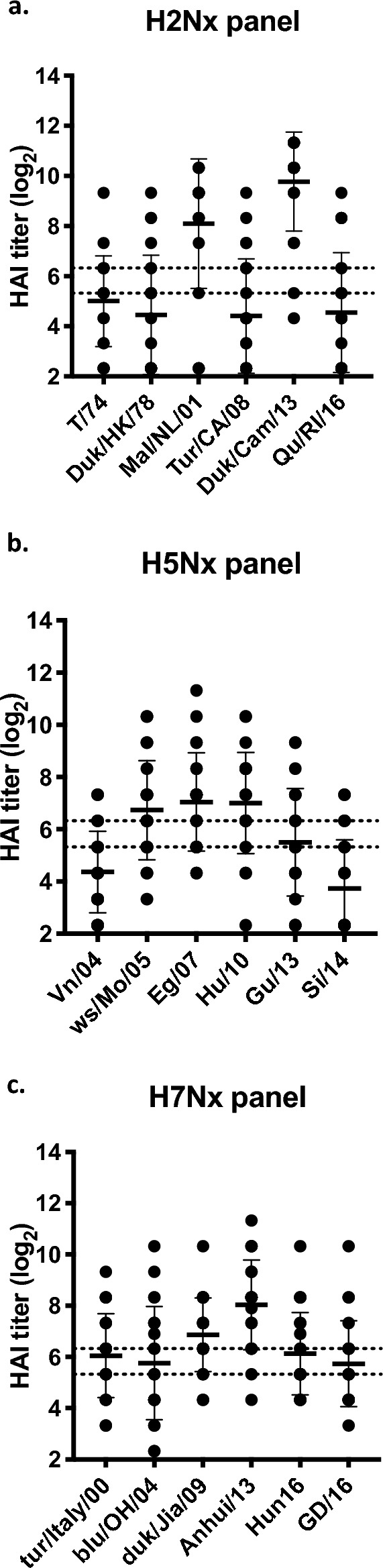
Serum HAI antibody titers for pre-pandemic influenza viruses after vaccination in mice. Immunologically naïve DBA/2J mice were vaccinated intramuscularly (i.m.) three times at four-week intervals with heptavalent formulations of COBRA recombinant HA and NA proteins, with AddaVax™ as adjuvant. Sera collected two weeks after final vaccination were used for HAI assay against a panel of (**a**) H2Nx, (**b**) H5Nx, and (**c**) H7Nx. The virus strains are listed along the x-axis. The y-axis indicates the log_2_ HAI titers for each vaccinated group and presents them as absolute mean values ± SEM. The dotted lines indicate HAI titers ranging from 1:40 (lower line) and 1:80 (upper line).

Mice vaccinated with the heptavalent COBRA HA vaccinations had antibodies with HAI activity against a wide breath of H2Nx VLPs (Fig. 2a), representing three different phylogenetic clades^17^. Antibodies had higher HAI activity against clade 1 viruses (Mal/NL/01 and Duk/Cam/13) compared to clade 2 viruses (T/64 and Duk/HK/78) and clade 3 viruses (Tur/CA/08 and Qu/RI/16). Mice vaccinated with COBRA vaccine had HAI activity that averaged ~ 1:40 or higher against strains from clade 2.2 (ws/Mo/05), subclade 2.2.1 (Eg/07), subclade 2.3.2.1 (Hu/10), and subclade 2.3.4.2 (Gu/13) (Fig. 2b). Antibody titers were lower against clade 1 (VN/04) and subclade 2.3.4.4 (Si/14) H5 viruses. COBRA vaccinations elicited antibody titers ≥ 1:40 for all H7Nx strains representing multiple lineages (Fig. 2c).

### Quadrivalent and heptavalent COBRA vaccinations elicit antibodies with NAI activity

**Figure 3:**
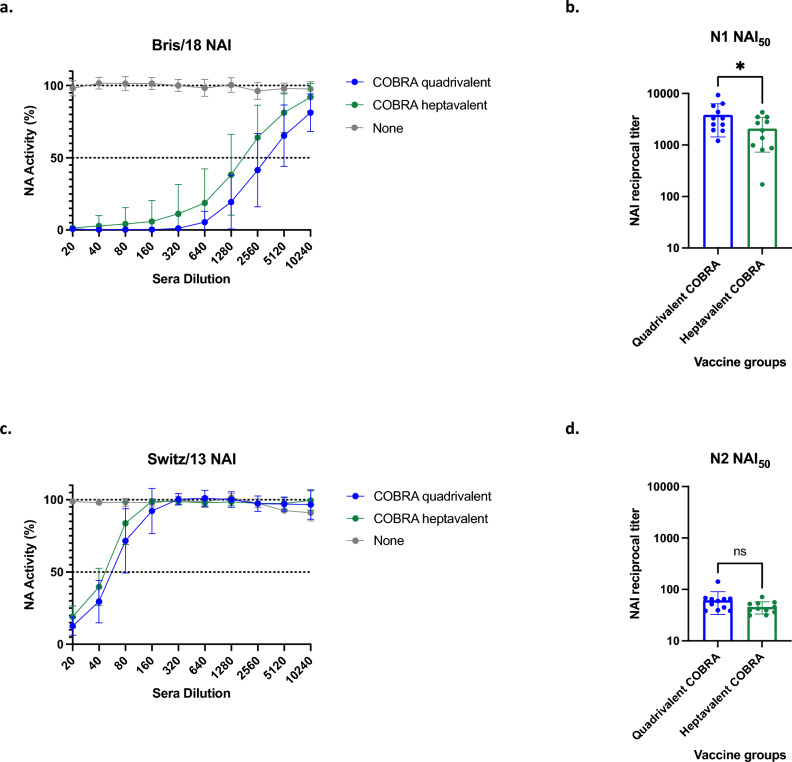
Serum NAI antibody titers after vaccinations in mice. Immunologically naïve DBA/2J mice were vaccinated intramuscularly (i.m.) three times at four-week intervals with quadrivalent (green) or heptavalent (blue) formulations of COBRA recombinant HA and NA proteins, with AddaVax™ as adjuvant. Sera collected two weeks after final vaccination were used for NAI assay against (**a**, **b**) N1 and (**c**, **d**) N2. Sera were diluted two-fold from starting dilution of 1:20 to quantify NAI titer (**a**, **c**). Non-linear regression was conducted from these results to obtain reciprocal titer that inhibited 50% of the NA activity (**b**, **d**). Statistical differences between NAI_50_ titers were analyzed by unpaired *t*-tests by GraphPad Prism 9 software (GraphPad, San Diego, CA, USA). A *p* value of less than 0.05 was defined as statistically significant (*, *p* < 0.05; **, *p* < 0.01; ***, *p* < 0.001; ****, *p* < 0.0001).

Sera from COBRA vaccinated mice were tested for NAI activity. Quadrivalent and heptavalent COBRA groups had robust NAI activity against N1 (Fig. 3a). Quadrivalent COBRA groups had significantly higher NAI_50_ titer compared to heptavalent (Fig. 3b). NAI activity against N2 was lower for both groups compared to N1 (Fig. 3c), with quadrivalent and heptavalent having similar NAI_50_ titers (Fig. 3d).

### Quadrivalent and heptavalent COBRA vaccinations elicit neutralizing antibodies against seasonal viruses in mice

**Figure 4:**
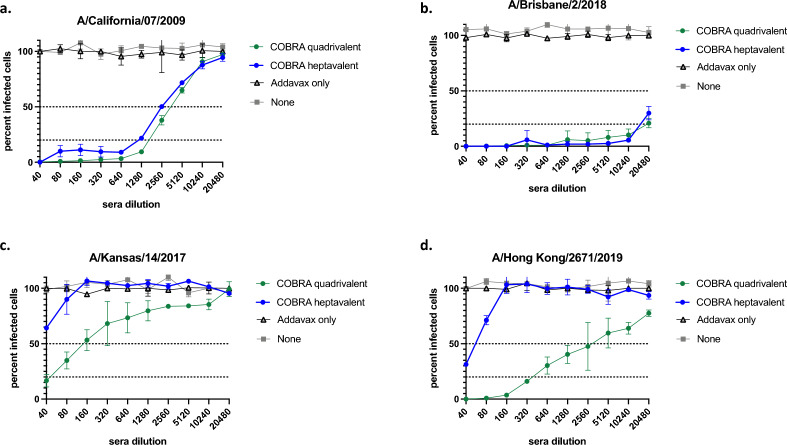
Neutralizing antibody titers for seasonal influenza viruses after vaccination in mice. Immunologically naïve DBA/2J mice were vaccinated intramuscularly (i.m.) three times at four-week intervals with quadrivalent (green line) or heptavalent (blue line) formulations of COBRA recombinant HA and NA proteins, with AddaVax™ as adjuvant. Sera collected two weeks after final vaccination were pooled for FRA against modern strains of (**a**, **b**) H1N1 and (**c**, **d**) H3N2 viruses. Top dashed line represents 50% neutralization, bottom dashed line represents 80% neutralization.

Neutralizing antibody activity was measured by focus reduction assay (FRA) against seasonal influenza viruses (Fig. 4). Heptavalent and quadrivalent COBRA vaccinated groups had similar levels of robust neutralizing activity against H1N1 strains (Fig. 4a,b). Both COBRA groups had higher neutralizing activity against Bris/18 compared to CA/09. Mice vaccinated with the quadrivalent COBRA HA vaccine had higher neutralization titers against the H3N2 strains compared to sera elicited by the heptavalent COBRA HA vaccine (Fig. 4c,d). Overall, neutralizing antibody titers correlated with HAI titers with the highest titers against H1N1 influenza viruses and lower titers against H3N2 strains.

### Quadrivalent and heptavalent COBRA vaccines protect mice against multiple subtypes of influenza

**Figure 5:**
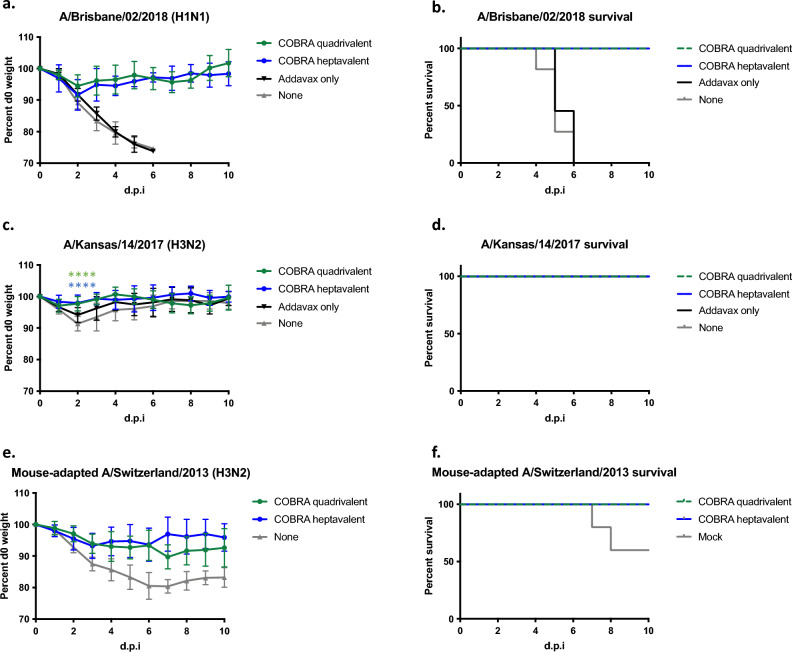
Protective efficacy against seasonal virus infection in vaccinated mice. Naïve DBA/2J mice (n = 11 per group) were vaccinated i.m. three times at four-week intervals with quadrivalent (green) or heptavalent (blue) formulations of COBRA recombinant HA and NA proteins, with AddaVax™ as adjuvant. Four weeks after final vaccination, mice were intranasally infected with (**a**, **b**) lethal dose of A/Brisbane/02/2018 (3 × 10^6^ PFU), (**c**, **d**) A/Kansas/14/2017 (6 × 10^6^ PFU), or (**e**, **f**) mouse-adapted A/Switzerland/2013 (1 × 10^5^ PFU). The animals were observed for clinical signs and their body weight was recorded daily post infection. Statistical differences after infection with H3N2 was analyzed by multiple unpaired t-tests by GraphPad Prism 9 software (GraphPad, San Diego, CA, USA). A *p* value of less than 0.05 was defined as statistically significant (*, *p* < 0.05; **, *p* < 0.01; ***, *p* < 0.001; ****, *p* < 0.0001).

Next, the ability of these vaccines to protect against viral challenge was determined (Fig. 5). Four weeks after final vaccination, mice were challenged with either an H1N1 (Bris/18) or H3N2 (KS/17) influenza virus. Mice vaccinated with the adjuvant only and negative control mice had a rapid drop in body weight following Bris/18 infection (Fig. 5a) and all mice reached humane endpoints by day 6 post-infection (Fig. 5b). All mice vaccinated with either set of the COBRA vaccines were protected with little weight loss and all mice survived lethal Bris/18 challenge (Fig. 5a,b). All mice, regardless of vaccination, survived KS/17 (H3N2) virus challenge (Fig. 5c,d) COBRA vaccinated groups had significantly less weight loss on day 2 post infection compared to adjuvant only and negative control mice (Fig. 5c). Subsequently, a mouse-adapted strain of H3N2 was used for challenge. Another set of mice were infected with mouse-adapted A/Switzerland/9715293/2013 (MA-Switz/13) (Fig. 5e,f). Mice given no vaccination lost a significant amount of weight, causing 40% mortality. Quadrivalent and heptavalent COBRA vaccinated mice lost 10% or less of original body weight (Fig. 5).

**Figure 6:**
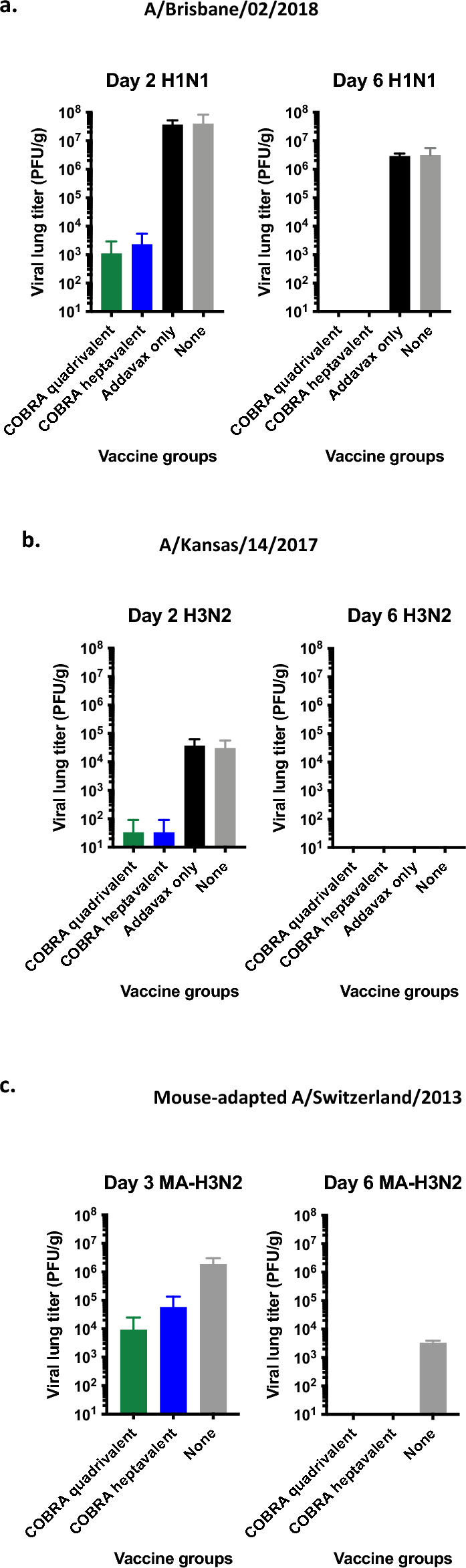
Lung viral titers after seasonal challenge. Lungs were taken on days 2 or 3 (n = 3) and day 6 (n = 3) post infection with Bris/18 (**a**), KS/17 (**b**), or MA_Switz/13 (**c**). Viral titers in lung tissues are presented as PFU/g on the y-axis.

Viral titers in lungs were quantified after seasonal virus infections. After Bris/18 infection, adjuvant only or negative control mice had high viral lung titers (~ 5 × 10^7^ pfu/g) on day 2 post-infection that remained high on day 6 post-infection (Fig. 6a). Mice vaccinated with either set of COBRA vaccines had viral lung titers that were 4 logs lower (~ 5 × 10^3^ pfu/g) than lung viral titers in mock vaccinated mice at day 2 post infection (Fig. 6a). There were no detectable lung viral titers in COBRA vaccinated mice on day 6. After KS/17 infection, mice vaccinated with adjuvant only or mock vaccinated mice had ~ 1 × 10^4^ pfu/g on day 2 post-infection, but COBRA vaccinated mice had less than 100 pfu/g (Fig. 6b). KS/17 virus was not detectable in the lungs of any mouse on day 6 post-infection. After MA-Switz/13 challenge, quadrivalent COBRA mice had ~ 1 × 10^4^ pfu/g lung viral titer on day 3 post infection, and heptavalent COBRA mice had ~ 5 × 10^4^ pfu/g on day 3 post infection. Negative control mice had lung viral titers that were 2 logs higher on day 3 post infection, ~ 2 × 10^6 ^pfu/g. By day 6 post infection, all COBRA vaccinated mice had no detectable vial lung titers, and negative control mice had ~ 10^3^ pfu/g.

**Figure 7:**
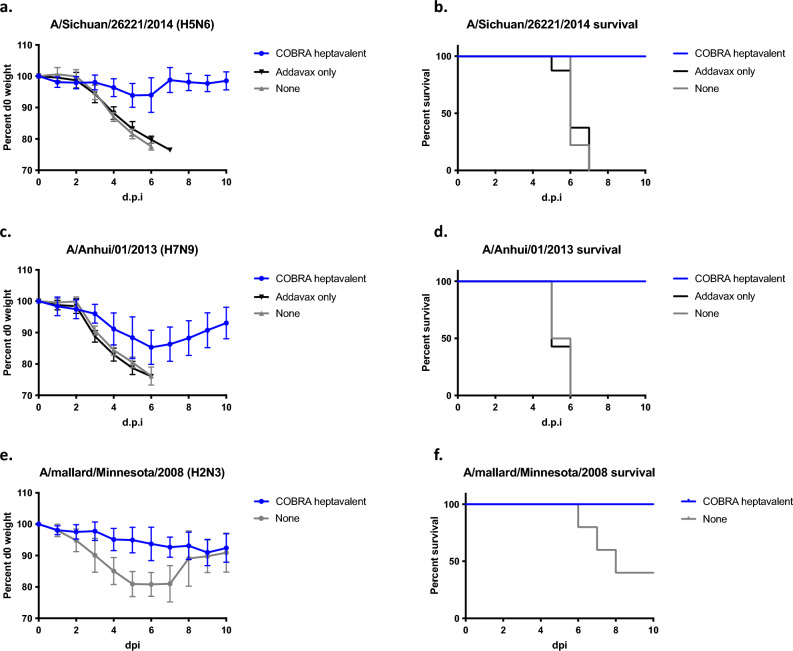
Protective efficacy against pre-pandemic virus infection in vaccinated mice. Immunologically naïve DBA/2J mice (n = 11 per group) were vaccinated i.m. three times at four-week intervals heptavalent (blue line) formulation of COBRA recombinant HA and NA proteins, with AddaVax™ as adjuvant. Four weeks after final vaccination, mice were intranasally infected with (**a**, **b**) lethal dose of A/Sichuan/26221/2014 (10^6^ PFU) or (**c**, **d**) lethal dose of A/Anhui/01/2013 (10^2^ PFU), or (**e, f**) A/mallard/Minnesota/AI08-3437/2008 (10^4^ PFU) in 50 µL volume. The animals were observed for clinical signs and their body weight was recorded daily post infection.

Heptavalent COBRA vaccinated mice were also challenged with pre-pandemic influenza viruses (Fig. 7). Four weeks after final vaccination, mice were infected with lethal dose of Sich/14 (H5N6). Mice vaccinated with the adjuvant only or mock vaccinated mice lost greater than 20% of their original body weight by day 6–7 post-infection (Fig. 7a). All mock vaccinated or adjuvant only vaccinated mice reached humane endpoints by days 5–7 post-infection with no mice surviving challenge. In contrast, mice vaccinated with the heptavalent COBRA vaccine lost little or no body weight and all mice survived Sich/14 challenge (Fig. 7b). A second set of heptavalent COBRA vaccinated mice were challenged with lethal dose of Anh/13 (H7N9) that lost ~ 10% body weight by day 6 post-infection, but then regained body weight throughout the course of the 14-day observation (Fig. 7c) with all mice surviving infection (Fig. 7d). All adjuvant only or negative control mice reached humane endpoints on days 5 and 6. A third set of mice were challenged with mal/MN/08 (H2N3) (Fig. 7e,f). All heptavalent COBRA vaccinated mice survived challenge, where as negative control mice had 60% mortality (Fig. 7e).

**Figure 8:**
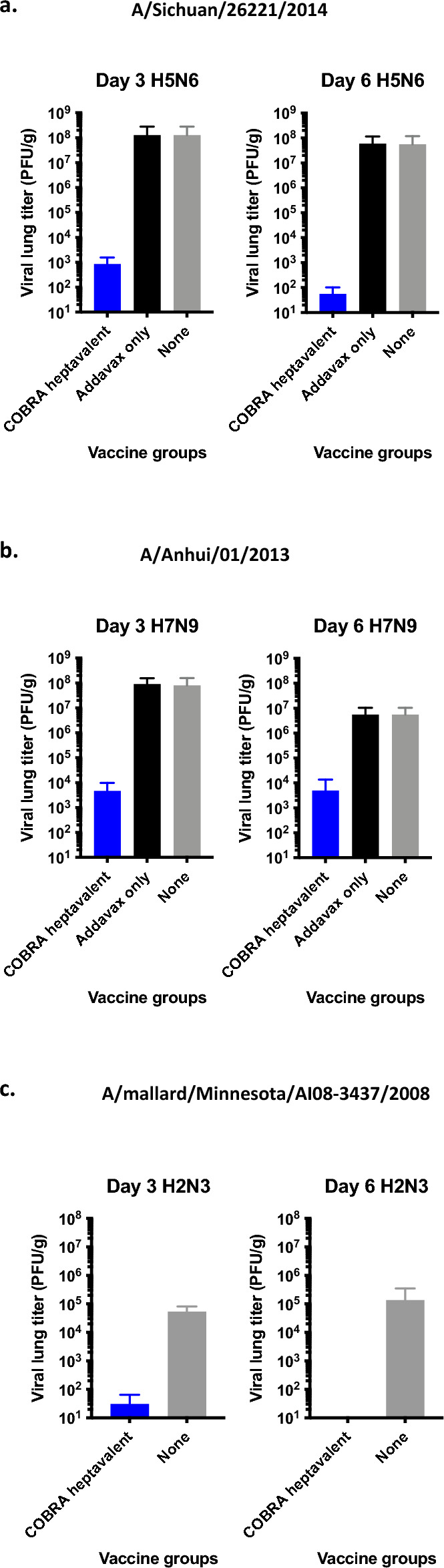
Lung viral titers after pre-pandemic challenge. Lungs were taken on day 3 (n = 3) and day 6 (n = 3) post infection with Sich/14 (**a**), Anhui/17 (**b**), or mal/MN/08 (**c**). Viral titers in lung tissues are presented as PFU/g shown on the y-axis.

Viral lung titers were quantified on days 3 and 6 after infection with pre-pandemic strains. After Sich/14 infection, adjuvant only or mock vaccinated mice had high viral lung titers (~ 10^6^–10^7^ pfu/g) on days 3 and 6 post-infection (Fig. 8a). Low viral lung titers (10^2^–10^3^ pfu/g) were detected in a few of the COBRA vaccinated mice on day 3 and 6 post-infection. Viral lung titers were detectable in all Anh/13 challenge mice on day 3 post-infection, however heptavalent COBRA vaccinated mice had viral titers that were 4 logs lower than mock or adjuvant only vaccinated mice (Fig. 8b). These viral lung titers declined in all mice by day 6 post-infection, but there were still high viral lung titers in adjuvant only or mock vaccinated mice (5 × 10^6^ pfu/g) (Fig. 8b). After H2N3infection, some heptavalent COBRA mice had detectable viral lung titers on day 3 (< 10^2^ pfu/g) and undetectable viral lung titers by day 6. Negative control mice had higher lung viral titers (~ 10^4^–10^5^ pfu/g) on day 3, which had not decreased by day 6.

## Discussion

Current influenza vaccines are composed of three to four seasonal strains that have to be updated annually^23,24^. It is difficult to predict the current circulating strain due to mutations in the antigenic surface glycoproteins (antigenic drift), resulting in vaccine mismatch and lower protective efficacy^25^. Pandemic outbreaks are even more difficult to predict and prepare. Often, random reassortment events between two or more influenza viruses (antigenic shift) create novel influenza A viruses against which humans have little to no immune protection^5^. Pre-pandemic influenza vaccine development and production are more costly and time consuming than development of seasonal influenza virus vaccines^11,26^. It is more effective to have pre-emptive vaccine strategies instead of reactive vaccine strategies for pre-pandemic viruses^27^.

Computationally optimized broadly reactive antigen (COBRA) methodology uses multi-layered consensus alignment of circulating strains to design an immunogen that reflects the antigenic evolution of the subtype while maintaining conserved epitopes^22^. Monovalent, single COBRA antigens elicited cross-protective immune responses compared to single wild type HA or NA counterparts^14–19^. The HA trimer is responsible for cell entry and membrane fusion^28^. Antibodies directed against HA neutralizes virus by inhibiting attachment and entry^29^. The NA tetramer has enzymic activity that releases newly formed virus from infected cells^30^. NA works in tandem with HA, resulting in effective infection and spread^31,32^. Prior to this study, multivalent combinations of COBRA HA combined with COBRA NA immunogens had not been tested in vivo. COBRA HA and NA immunogens were selected to represent different subtypes. Each COBRA immunogen selected for this study has previously demonstrated to elicit wider breadth of antibody responses than wild type comparators and other previously designed COBRA constructs (Table 1).

In this study, quadrivalent and heptavalent COBRA vaccinations provided robust protective antibody responses against many IAV subtypes. Both of these vaccine formulations elicited similar broadly reactive HAI antibodies against a panel of H1N1 and H3N2 influenza strains (Fig. 1). In addition, COBRA heptavalent vaccines elicited HAI responses against pre-pandemic subtypes H2Nx, H5Nx, and H7Nx, with antibody binding spanning multiple clades for each subtype (Fig. 2). These results demonstrate that a large number of COBRA antigens can be administered together to increase breadth without reduction in antibody titers.

For H1 HAIs, both vaccines elicited robust antibody response against A(H1N1)2009 pandemic strains (CA/09, Mich/15, Bris/18, GD/19) (Fig. 1a,b). There was no reactivity to historical H1 strains (SI/86, Bris/07). Our group has previously designed COBRA immunogens which elicited HAI response against historic strains in mice^33^. However, since the historic strains have been completely replaced by the A(H1N1)2009 lineage^34^, we utilized the Y2 COBRA which are based on strains that are currently circulating^15^. The design of the Y2 COBRA was based on strains from 2016 to 2019 (Table 1), which may explain the increased neutralization titers for Bris/18 compared to CA/09 (Fig. 4a,b)*.* Currently circulating H1 viruses are more similar to Bris/18 compared to CA/09^34^.

Our group tested monovalent NA COBRA vaccines and demonstrated breadth and efficacy of protection^19,35^. In this study, we showed that multivalent COBRA vaccinations which included COBRA N1 and N2 elicited antibodies that inhibited NA activity against N1 and N2 (Fig. 3). Recombinant proteins were generated by our lab and used for NAI assays, since using wild type virus would cause steric hindrance by HA antibodies in the mouse sera and resulting in non-specific NA inhibition^36,37^.

Quadrivalent and heptavalent COBRA vaccinated mice were protected against seasonal IAV challenges (Fig. 5). Both COBRA vaccine groups were protected against lethal H1N1 challenge and had lower viral lung titers compared to mock vaccinated mice (Fig. 5b, 6a). Wild-type seasonal H3N2 influenza viruses do not replicate well in mice^38^, thus all mice challenged with KS/17 survived, including control groups (Fig. 5c,d). COBRA vaccinated mice had less weight loss (significant on day 2 post challenge) and decreased viral lung titers compared to adjuvant only and mock vaccinated mice (Fig. 6b), despite a titer < 1:40 HAI against KS/17. A mouse-adapted strain of H3N2 (MA-Switz/13) was subsequently used in order to better demonstrate vaccine protection. Both COBRA vaccine groups were completely protected against infection, while 40% of mice given no vaccination reached humane endpoints (Fig. 5e,f).

The correlate of protection elicited by seasonal influenza vaccines in humans has historically been associated with an HAI titer of 1:40 or greater^39^. In this study, as well as others, the 1:40 HAI titer does not necessarily correlate with protection against pre-pandemic potential from subtypes such as H2, H5, and H7. In addition, recent human infections studies indicate that anti-HA stem and anti-NA antibodies elicit protective immunity independent of hemagglutination inhibition of receptor binding^20,40,41^. HA stem-based antibodies are more conserved and cross reactive compared to HA head binding antibodies^42,43^. Anti-stem HA antibodies can aid in neutralization by inhibiting fusion^44^ and has antiviral activity via Fc-receptor mediated antibody-dependent cellular cytotoxicity (ADCC)^45^. Anti-NA antibodies can reduce the spread of virus by inhibiting release of viral progeny^46^.

NA vaccines have not been as extensively examined compared to HA^47^. NA is considered to be immunosubdominant to HA after infection and vaccination^4,48^. There are more HA molecules present on the surface of the virus compared to NA^49^, contributing to higher levels of anti-HA antibodies after natural infection^50^. Unlike HA, inactivated influenza virus vaccines have varied amounts of NA in the composition that is not standardized between production lots^51^. However, vaccinating with monovalent COBRA NA reduced lung viral titers in vaccinated mice compared to controls following influenza virus infection^19^. Combining NA in an influenza vaccine would complement HA, since antigenic drift of HA and NA are independent of each other^52^, and NA has lower mutation rates than HA^53^.

COBRA heptavalent vaccinations elicited broad breadth of antibody responses against pre-pandemic subtypes H2, H5, and H7 (Fig. 2). Recent human infections of avian H5 and H7 are causes for concern^54^. Highly pathogenic H5N1 avian flu virus emerged in 1996 and have caused outbreaks in over 60 countries and infected over 800 humans with a fatality rate of almost 53%^55,56^. Since 2013, there have been six H7N9 outbreaks with over 1500 confirmed infections and over 600 deaths^57,58^. Heptavalent COBRA vaccinations protected mice from lethal doses of viruses with pandemic potential. The H5 challenge virus in this study (Si/14) is from subclade 2.3.4.4, which recently had an abrupt and widespread global distribution^59^. It originated from the highly pathogenic H5 strain A/goose/Guangdong/1/1996 (gs/GD/96, clade 2.3.4) that has been responsible for hundreds of deaths since its emergence in 1997^60^. All vaccinated mice survived lethal H7 Anh/13 infection as well. Anh/13 virus was not cleared as quickly as the other three infections, which is indicated by the slower recovery in weight.

The current wave of highly pathogenic avian influenza (HPAI) H5N1 infection in wild birds and poultry have caused global alarm^55^. Since 2020, this virus from the 2.3.4.4b subclade has overtaken other circulating H5Nx subtypes, causing outbreaks in Asia, Europe, Africa, and North America^61–63^. Currently, this is the largest of the three H5Nx viral outbreaks since 2005 resulting in ~ 200 million poultry deaths by the end of 2022^64^. This wave is infecting not only wild birds and domestic poultry, but marine mammals as well^61,65,66^ with a human mortality in Cambodia in 2023. Transmission of H5Nx to humans is not completely understood, but change in sialic receptor preference from α-2,3 to α-2,6^67^ and six mutations in HA protein (Q222L, G224S, E186D, K189R, S223N, and N182K)^68^ are linked to infectivity of human cells in the upper respiratory tract. In the past decade, human infections have been from subclades 2.2.1 and 2.3.4.4 viruses^59,69,70^. In February 2023, it was reported that two human H5N1 (subclade 2.3.2.1c) infections occurred in Cambodia^71^. COBRA heptavalent vaccination elicited antibodies that recognized viruses representing these clades-Eg/07 (subclade 2.2.1), Sich/14 (subclade 2.3.4.4), and Hu/10 (subclade 2.3.2.1), as well as viruses from other clades.

Influenza virus is constantly mutating, making global circulation patterns difficult to predict. The COVID-19 pandemic significantly reduced influenza circulation during the 2020–2021 and 2021–2022 seasons, lowering global exposure and immunity. However, following intense COVID-19 vaccination campaigns, influenza cases increased dramatically during the 2022–2023 influenza season, peaking earlier than historical norms, which led to increased disease severity^72^. Current vaccines are only effective when well-matched to the circulating strains^73^. The COBRA methodology can capture the antigenic evolution, while maintaining conserved epitopes to protect against past viruses that may re-emerge, currently circulating viruses, and potential future viruses^74^. The COBRA immunogen can be implemented in many different delivery platforms, such as mRNA and viral vectors. Other influenza virus vaccines use a large number of antigens, up to twenty^75,76^, in order to elicit protective immune responses against both seasonal and pre-pandemic strains. Due to the breadth of protection from each individual immunogen, COBRA-based HA and NA vaccines would most likely not need as many components included in an influenza virus vaccine.

Next steps will include IBV COBRA HA in the formulation to assess efficacy against both IAV and IBV. Seasonal H3 viruses mutate at a higher rate than H1 or IBV^77^, with two antigenically distinct clades circulating. Including more than one COBRA H3 HA proteins with elicitation of distinct antibody profiles in the vaccine formulation may increase breadth of antibody activity against H3N2 drifted viruses. Since most people have been previously exposed to seasonal influenza viruses, future studies in pre-immune ferrets or humans may reveal the true effectiveness of COBRA based vaccine strategies^78^. These future studies can confirm that multivalent COBRA HA and NA formulation is a promising candidate for a broadly-reactive influenza virus vaccine.

## Methods

### Influenza viruses and virus-like particles

Influenza viruses were obtained through either the Influenza Reagents Resource (IRR), BEI Resources, the Centers for Disease Control (CDC), or provided by Virapur (San Diego, CA, USA). Viruses were passaged once in the same growth conditions as they were received, either embryonated chicken eggs or semi-confluent Madin-Darby canine kidney (MDCK) cell cultures as per the instructions provided by the World Health Organization (WHO)^79^.

H1N1 virus lots were titered with 0.8% turkey erythrocytes and made into aliquots for single use applications. H3N2 virus lots were titered with 0.75% guinea pig erythrocytes in the presence of 20 nM Oseltamivir, and made into aliquots for single-use applications. H5Nx virus lots were tittered with 1% horse erythrocytes and made into aliquots for single use applications.

H1N1 Influenza viruses used in this study include: A/Solomon Islands/03/2006 (SI/06), A/Brisbane/59/2007 (Bris/07), A/California/07/2009 (CA/09), A/Michigan/45/2015 (Mich/15), A/Brisbane/02/2018 (Bris/18), and A/Guangdong-Maonan/SWL1536/2019 (GD/19).

H3N2 Influenza viruses used in this study include: A/Panama/2007/1999 (Pan/99), A/Switzerland/9715293/2013 (Switz/13, clade 3c.3a); mouse-adapted Switz/13 (MA-Switz/13); A/Hong Kong/4801/2014 (HK/14, clade 3c.2a); A/Singapore/IFNIMH-16-0019/2016 (Sing/16, clade 3c.2a1); A/Kansas/14/2017 (KS/17, clade 3c.3a); A/South Australia/34/2019 (SA/19, clade 3c2.a1b/131K); A/Hong Kong/2671/2019 (HK/19, clade 3c2.a1b/137F).

H5Nx viruses used for this study were PR8 reassortant viruses containing internal genes from the mouse adapted strain A/Puerto Rico/8/1934: A/Vietnam/1203/2004 (Vn/04; H5N1), A/whooper swan/Mongolia/244/2005 (ws/Mo/05; H5N1), A/Egypt/321/2007 (Eg/07; H5N1), A/Hubei/1/2010 (Hu/10; H5N1), A/Guizhou/1/2013 (Gu/13; H5N1), A/Sichuan/26221/2014 (Si/14; H5N6). H7N9 virus used was A/Anhui/01/2013 (Anhui/13; H7N9). For H2Nx and H7Nx HAIs, virus like particles (VLPs) were used instead of BSL-3 level viruses. VLPs were produced as previously described^17,80^. Briefly, HEK 293T cells were transiently transfected with plasmids expressing HIV-1 p55 Gag sequence, NA (A/South Carolina/1/1918 H1N1 for H2 VLPs, A/Thailand/1(KAN-1)/2004 H5N1 for H7 VLPs), and various wild type H2 or H7 HAs. The cells were incubated for 72 h at 37 °C. Supernatant was collected and centrifuged at low speed and filtered through 0.22 µm sterile filter. Next, VLPs were purified via ultracentrifugation on a 20% glycerol cushion (23,500 g for H2, 100,000 g for H7) for 4 h at 4 °C. Pellets were resuspended in PBS and stored in − 80 °C until use.

For H2 HAIs, VLPs produced were: A/Taiwan/1/1964 (T/64), A/Duck/Hong Kong/273/1978 (Duk/HK/78), A/Mallard/Netherlands/13/2001 (mal/NL/01), A/Turkey/California/1797/2008 (Tur/CA/08), A/Duck/Cambodia/ 419W12M3/2013 (Duk/Cam/13), A/quail/Rhode Island/2016 (Qu/RI/16). For H7 HAIs, VLPs produced were: A/turkey/Italy/589/2000 (tur/Italy/00), A/blue-winged teal/Ohio/658/2004 (blu/OH/04), A/duck/Jiangxi/3230/2009 (duk/Jia/09), A/Anhui/01/2013 (Anhui/13), A/Hunan/02650/2016 (Hun/16), A/Guangdong/17SF003/2016 (GD/16). A/Mallard/Minesota/AI08-3437/2008 (mal/MN/08, H2N3) virus was used for challenge.

### Vaccine design and production

Computationally optimized broadly reactive antigen (COBRA) methodology used to produce influenza HA and NA recombinant proteins representing seasonal and pandemic subtypes. The design and production of these COBRA antigens have been previously published by our lab (Table 1). Briefly, full length wild-type (WT) HA or NA sequences were downloaded from Global Initiative on Sharing Avian Influenza Data (GISAID) for each subtype. The sequences were aligned and primary consensus sequences were created from clusters of WT sequences based on percent similarity. Secondary consensus sequences were created from the primary sequences; this multi-layered consensus building way continued until a final COBRA sequence was obtained.

The production of recombinant COBRA HA and NA proteins has been previously described^81^. Briefly, each COBRA HA or NA nucleotide sequence was cloned into a pcDNA3.1 + plasmid vector, being truncated by removing the transmembrane domain and replacing with a T4 fold-on domain, an AviTag, and a 6 × His tag for purification by immobilized metal affinity chromatography. The plasmid was transfected into human endothelial kidney 293T (HEK 293T) suspension cells for protein expression. Protein concentration of the purified soluble proteins were determined by bicinchoninic acid assay (BCA) according to the manufacturer’s instructions (Thermo Fisher, Waltham, MA).

### Mouse vaccination and infection

DBA/2J mice (females, 6–8 weeks old) were purchased from The Jackson Laboratory (Bar Harbor, ME, USA). The mice were housed in microisolator units, and allowed free access to food and water. All animals were cared for under the USDA guidelines for laboratory animals, and all procedures were approved by the University of Georgia Institutional Animal Care and Use Committee (IACUC) (no. A2018 06-018-Y3-A16). The mice were randomly divided into four vaccine groups (n = 44) and each vaccine group had four different infection groups (n = 11). The vaccine groups were quadrivalent COBRA formulation, consisting of seasonal components H1 HA (Y2), H3 HA (NG2), N1 NA (N1I), and N2 NA (N2A); heptavalent COBRA formulation, consisting of seasonal Y2, NG2, N1I, N2A along with pandemic components H2 HA (Z1), H5 HA (IAN 8), and H7 HA (Q6); adjuvant only; or negative control. The vaccines were formulated with 3 µg of each recombinant protein or phosphate-buffered saline (PBS, Corning, Tewkbury, MA, USA) and adjuvanted with an emulsified squalene-based oil-in-water emulsion adjuvant, AddaVax™ (InvivoGen, San Diego, CA, USA) at 1:1 ratio. Vaccines were administered intramuscularly (i.m.) into the hind leg of the animals on day 0, 28, and 56 in a homologous prime-boost-boost regimen. Blood was collected from the facial vein 14 days following each vaccination, on day 14, 42, and 70. Sera was isolated from the blood by centrifugation at 5000 rpm for 5 min in BD Microtainer blood collection tubes (BD, Franklin Lakes, NJ). Clarified serum was removed and frozen at − 20 °C.

Four weeks after final vaccination, mice were challenged intranasally (i.n.) with 50 µL volume of live influenza virus: lethal dose of Bris/18 (3 × 10^6^ PFU), KS/17 (6 × 10^6^ PFU), MA-Switz/13 (1 × 10^5^ PFU), Mal/MN/08 (1 × 10^4^ PFU), lethal dose of H5N6 Sich/14 (10^6^ PFU), or lethal dose of Anhui/13 (10^2^ PFU, under biosafety level 3 (BSL3) conditions). Following infection, the animals’ body weights were recorded daily, and the animals were monitored twice daily for clinical signs (labored breathing, lethargy, hunched back, ruffled fur, failure to respond to stimuli, and severe respiratory distress) for 14 days. Weight loss more than 25% was used as a primary measurement for determination of humane endpoint. On days 2 or 3 and 6 after infection, three animals from each group were sacrificed, and lungs were collected to assess viral load. Lungs were frozen on dry ice and stored at − 80 °C. Remaining mice (n = 5) were euthanized 14 days after challenge.

All animal procedures were approved by the University of Georgia Institutional Animal Care and Use Committee (IACUC) (no. 2018 06-018-Y3-A16) and performed in accordance with the National Research Council (NRC) Guide for the Care and Use of Laboratory Animals, the Animal Welfare Act, and the CDC/NIH Biosafety in Microbiological and Biomedical Laboratories. This study was reported in accordance with the ARRIVE guidelines^82^.

### Hemagglutination-inhibition (HAI) assay

The hemagglutination inhibition (HAI) assay was used to assess functional antibodies to the HA that are able to inhibit agglutination of turkey erythrocytes for H1N1 viruses and H2, H7 VLPs. Horse erythrocytes were used for PR8 reassortant H5 viruses. Guinea pig erythrocytes were used for H3N2 viruses. Guinea pig red blood cells are frequently used to characterize contemporary A(H3N2) influenza strains that have developed a preferential binding to α2,6-linked sialic acid receptor^83^. The protocols were adapted from the WHO laboratory influenza surveillance manual^1^. To inactivate nonspecific inhibitors, sera samples were treated with receptor-destroying enzyme (RDE) (Denka Seiken, Co., Japan) prior to being tested. Briefly, three parts of RDE was added to one part of sera and incubated overnight at 37 °C. RDE was inactivated by incubation at 56 °C for 30 min.

RDE-treated sera were diluted in a series of two-fold serial dilutions in v-bottom microtiter plates. An equal volume of each virus or VLP, adjusted to approximately 8 hemagglutination units (HAU)/50 µl was added to each well. The plates were covered and incubated at room temperature for 20 min, and then 0.8% turkey erythrocytes (or 1% horse erythrocytes for H5) (Lampire Biologicals, Pipersville, PA, USA) in PBS were added. Prior to use, the red blood cells (RBCs) were washed twice with PBS, stored at 4 °C, and used within 24 h of preparation. The plates were mixed by gentle agitation, covered, and the RBCs were allowed to settle for 30 min (or 1 h for H5) at room temperature. The HAI titer was determined by the reciprocal dilution of the last well that contained non-agglutinated RBCs. Positive and negative serum controls were included for each plate.

Similarly, for H3N2 HAI, RDE-treated sera were diluted in a series of two-fold serial dilutions in v-bottom microtiter plates. An equal volume of each H3N2 virus, adjusted to approximately 8 HAU/50 µl in the presence of 20 nM Oseltamivir carboxylate, was added to each well. The plates were covered and incubated at room temperature for 30 min, and then 0.75% guinea pig erythrocytes (Lampire Biologicals) in PBS were added. Prior to use, the RBCs were washed twice with PBS, stored at 4 °C, and used within 24 h of preparation. The plates were mixed by gentle agitation, covered, and the RBCs were allowed to settle for 1 h at room temperature. The HAI titer was determined by the reciprocal dilution of the last well that contained non-agglutinated RBCs. Positive and negative serum controls were included for each plate.

All mice were negative (HAI ≤ 1:10) for pre-existing antibodies to human influenza viruses prior to infection or vaccination, and for this study, a positive HAI reaction (HAI +), or “sero-protection”, is defined as an HAI titer ≥ 1:40, and “seroconversion” refers to a fourfold increase in titer compared to baseline, as per the WHO and European Committee for Medicinal Products to evaluate influenza vaccines^84^.

### Focus reduction assay

Focus reduction assay (FRA) was used the assess the ability of polyclonal sera from vaccinated mice to neutralize H1N1 and H3N2 viruses in vitro, as previously described^85^. Briefly, MDCK-SIAT1 cells were plated at 5 × 10^4^ cells per well in a 96-well plate in media (DMEM containing 5% heat-inactivated fetal bovine serum and penicillin–streptomycin). RDE-treated mouse sera were serially diluted twofold starting at 1:20 dilution in virus growth medium (DMEM containing 0.1% BSA, penicillin–streptomycin, and 1 mg/mL TPCK-treated trypsin). 50 mL of sera were added to the cell monolayers. Afterward, 50 µL of virus (600 focus forming units (FFU)/50 µL) were added and the plates incubated for 2 h at 37 °C with 5% CO_2_. The cells were overlaid with 1.2% Avicel (FMC Health and Nutrition, Philidelphia, PA) in 2 × modified Eagle medium containing 0.1% BSA, penicillin–streptomycin, and 1 µg/mL TPCK-treated trypsin. Plate were incubated 24 h at 37 °C with 5% CO_2_. The overlays were removed and the cell monolayers washed with PBS to remove any residual Avicel. The plates were fixed with 4% formalin and cells were permeablized with 0.5% Triton X-100 in PBS/glycine. The plates were washed with PBS + 0.1% Tween 20 and incubated for 1 h at room temperature with monoclonal antibody against influenza A or B nucleoprotein from IRR. After washing three times, the cells were incubated with goat anti-mouse peroxidase labeled IgG (474-1802; SeraCare, Inc, Milford, MA) for 1 h at room temperature. The plates were washed three times and infectious foci were visualized by adding TrueBlue substrate (SeraCare) containing 0.03% H_2_O_2_ to the cells for 10 min at room temperature. The reaction was stopped by washing with distilled water five times. The foci were counted using BioSpot analyzer with ImmunoCapture 6.4.87 software (CTL, Shaker Heights, OH). The virus control well containing no sera was used for comparison of focus reduction.

### Neuraminidase inhibition (NAI) assay

In order to assess NAI activity from polyclonal mouse sera, recombinant NA proteins representing wild type strains were generated in our lab as an alternative to using whole virus. This was done to prevent HA binding antibodies in the sera from inhibiting NA activity via steric hindrance^36,37^. Bris/18 (N1) and Switz/13 (N2) soluble tetrameric NA proteins were produced as previously described^81^. Briefly, NA nucleotide sequences were cloned into a pcDNA3.3-TOPO vector (Thermo Fisher Scientific), transiently transfected into EXPI293F cells (Thermo Fisher Scientific) following the ExpiFectamine 293 transfection kit protocol, and purified by immobilized metal affinity chromatography. Protein concentrations determined by BCA. NA acitivty was determined by enzyme-linked lectin assay (ELLA) as previously described^19^: high-affinity Immunoblot 4HBX 96-well flat bottom plates (Thermo Fisher Scientific) were coated with 100μL of 25μL/mL fetuin (Sigma-Aldrich, St. Louis, MO) in commercial KPL coating buffer (Seracare Life Sciences, Milford, MA, USA) overnight at 4 °C. Plates were washed 3× in PBS-T (PBS + 0.05% Tween 20) and serial dilutions of rNA in sample diluent (Dulbecco’s phosphate-buffered saline containing 0.133 g/L CaCl_2_ and 0.1 g/L MgCl_2_ (DPBS) + 1% BSA + 0.5% Tween 20) were added to the plates and incubated for 17 h at 37 °C. Plates were washed 6×, and 100 µL of peanut agglutinin-HRPO (Sigma-Aldrich) diluted 1,000 fold in conjugate diluent (DPBS + 0. 1% BSA) was added. Plates were incubated for 2 h at RT. Plates were washed 3× and 100 µL of 500 µg/mL of o-phenylenediamine dihydrchloride (OPD) in 0.05 M phosphate-citrate buffer with 0.03% sodium perborate pH 5.0 (Sigma-Aldrich) was added for 10 min at RT in the dark. 100 µL of 1N sulfuric adic was added to stop the reaction. The absorbance was read at 490 nm using the Biotek spectrophotometer (Epoch 2; Agilent Technologies, Santa Clara, CA, USA). Linear regression analysis was used to determine the concentration of rNA to use in NAI assay (90–95% NA activity).

To analyze NAI activity of polyclonal sera after vaccination, 50 µL two-fold serial dilutions of vaccinated mouse sera in sample diluent was combined with 50 µL rNA antigen (in the optimal concentration determined above). This mixture was placed in 96-well fetuin-plates (previously coated overnight then washed 3×) and incubated for 17 h at 37 °C. Plates were washed 6× and incubated with 100 µL peanut agglutinin-HRPO for 2 h at RT. Plates were washed 3× and 100µL OPD added for 10 min at RT in the dark. 100 µL 1N sulfuric adic was added to stop the reaction. Plates were read at 490 nm on the Biotek spectrophotometer. NA percent activity was determined by substracting the mean background absorbance of the negative-control wells and dividing the serum absorbance by the mean virus positive-control wells muiltipled by 100. Nonlinear regression analysis was used to determine 50% NAI titer on GraphPad Prism.

### Influenza viral plaque assay

For mouse lung plaque assay, MDCK cells (Sigma, St. Louis, MO, USA) were seeded into each well of a six-well plate at a concentration of 1 × 10^6^ cells/well one day prior to performing the plaque assay. On the day of the assay, frozen lung tissues were thawed on ice were weighed and homogenized in 1 ml of DMEM (Thermo Fisher, Waltham, MA, USA). The homogenate was centrifuged at 2000 rpm for 5 min to remove tissue debris, and the supernatant was collected and subjected a serial tenfold dilution in DMEM supplemented with 1% penicillin–streptomycin (DMEM + P/S) (Thermo Fisher, Waltham, MA, USA). When MDCK cells reached 90% confluency in each well, the plates were washed 2× with DMEM + P/S, and infected with 100 µL of each dilution of homogenate supernatant. The plates were then shaken every 15 min for 1 h. After 1 h of incubation, the supernatant was removed and cells were washed twice with fresh DMEM + P/S. Following the second wash, a solution of 2× MEM and 1.6% agarose (Thermo Fisher, Waltham, MA, USA) mixed 50:50 v/v, and supplemented with 1 µg/mL of TPCK trypsin (Thermo Fisher, Waltham, MA, USA) was added into each well. Plates were then incubated at 37 °C + 5% CO_2_ for 72 h. After 72 h, the gel overlays were removed from each well, and the cells were fixed with 10% buffered formalin for 10 min and stained with 1% crystal violet (Thermo Fisher, Waltham, MA, USA) for 10 min at room temperature. Plates were then rinsed thoroughly 5× with fresh water to remove excess crystal violet. Plates were allowed to air dry for 24 h and the viral plaques were enumerated as the reciprocal of each dilution. The lung viral titers were calculated and presented as plaque forming units (PFU)/g of lung tissue.

### Statistical analysis

Data is presented as absolute mean values ± standard error of the mean (SEM). One-way ANOVA, unpaired multiple *t* tests, and two-way ANOVA with multiple comparisons were used to analyze the statistical differences between vaccine groups using GraphPad Prism 9 software (GraphPad, San Diego, CA, USA). A “*p*” value less than 0.05 was defined as statistically significant (*, *P* < 0.05; **, *P* < 0.01; ***, *P* < 0.001; ****, *P* < 0.0001).

## Data Availability

The data that support the findings of this study are available from the corresponding author upon reasonable request.
